# Ipecac as a Hæmostatic

**Published:** 1878-10

**Authors:** 


Ipecac as a Hæmostatic.—I have administered ipecac as a hæmostatic in hæmoptysis frequently for years past, and have found it the most efficient remedy in such cases. I have also used for the same purpose ergot and pyrogallic acid, both of which are efficient remedies, in hæmoptysis as well as in post-partum hemorrhage. But ipecac is certainly the best remedy in the treatment of hemoptysis; and comparatively limited experience in its use in the treatment of post-partum hemorrhage leads me to believe that it is a too-much-neglected medicine in such cases. As a hæmostatic ipecac is an *old* remedy, but of all its wonderful power and efficiency in this direction my own opinion fully corroborates that of the great clinicien, Trousseau, to whose writings in this connection I would further refer.—Dr. Q. C. Smith, in the *Pacific Medical and Surgical Journal.*

